# Altered mRNA Splicing, Chondrocyte Gene Expression and Abnormal Skeletal Development due to *SF3B4* Mutations in Rodriguez Acrofacial Dysostosis

**DOI:** 10.1371/journal.pgen.1006307

**Published:** 2016-09-13

**Authors:** Felipe Marques, Jessica Tenney, Ivan Duran, Jorge Martin, Lisette Nevarez, Robert Pogue, Deborah Krakow, Daniel H. Cohn, Bing Li

**Affiliations:** 1 Programa de Pós-Graduação em Ciências Genômicas e Biotecnologia, Universidade Católica de Brasília, Brasília, Brazil; 2 Laboratório de Biotecnologia, Universidade CEUMA, Campus Renascença, São Luís-MA, Brazil; 3 Department of Biomedical Sciences, Cedars-Sinai Medical Center, Los Angeles, California, United States of America; 4 Department of Pediatrics, Division of Genetics, University of California Los Angeles, Los Angeles, California, United States of America; 5 Department of Orthopaedic Surgery, University of California Los Angeles, Los Angeles, California, United States of America; 6 Department of Molecular, Cell, and Developmental Biology, University of California Los Angeles, Los Angeles, California, United States of America; 7 Department of Obstetrics and Gynecology, University of California at Los Angeles, Los Angeles, California, United States of America; 8 Department of Human Genetics, University of California Los Angeles, Los Angeles, California, United States of America; Murdoch Childrens Research Institute, AUSTRALIA

## Abstract

The acrofacial dysostoses (AFD) are a genetically heterogeneous group of inherited disorders with craniofacial and limb abnormalities. Rodriguez syndrome is a severe, usually perinatal lethal AFD, characterized by severe retrognathia, oligodactyly and lower limb abnormalities. Rodriguez syndrome has been proposed to be a severe form of Nager syndrome, a non-lethal AFD that results from mutations in *SF3B4*, a component of the U2 small nuclear ribonucleoprotein particle (U2 snRNP). Furthermore, a case with a phenotype intermediate between Rodriguez and Nager syndromes has been shown to have an *SF3B4* mutation. We identified heterozygosity for *SF3B4* mutations in Rodriguez syndrome, confirming that the phenotype is a dominant disorder that is allelic with Nager syndrome. The mutations led to reduced SF3B4 synthesis and defects in mRNA splicing, primarily exon skipping. The mutations also led to reduced expression in growth plate chondrocytes of target genes, including the *DLX5*, *DLX6*, *SOX9*, and *SOX6* transcription factor genes, which are known to be important for skeletal development. These data provide mechanistic insight toward understanding how *SF3B4* mutations lead to the skeletal abnormalities observed in the acrofacial dysostoses.

## Author Summary

The acrofacial dysostoses (AFD) are inherited disorders with abnormalities of the facial and limb bones. Rodriguez syndrome is a severe type of AFD that is usually lethal in the immediate perinatal period. Rodriguez syndrome has been proposed to be a severe form of Nager syndrome, a non-lethal AFD that results from mutations in *SF3B4*, a component of mRNA splicing machinery needed for proper maturation of primary transcripts. Furthermore, a case with a phenotype intermediate between Rodriguez and Nager syndromes has been shown to have an *SF3B4* mutation. We found that mutations in *SF3B4* produce Rodriguez syndrome, further demonstrating that it is allelic with Nager syndrome. The consequences of the mutations include abnormal splicing and reduced expression in growth plate chondrocytes of genes that are important for proper development of the skeleton, providing mechanistic insight toward understanding how *SF3B4* mutations lead to the skeletal abnormalities observed in the acrofacial dysostoses.

## Introduction

The acrofacial dysostoses (AFD) are a genetically heterogeneous group of inherited disorders unified by craniofacial and limb abnormalities. At least 18 types of AFD have been characterized and, depending on the specific patterns of limb abnormalities, they have been further classified into those with preaxial limb abnormalities, those with postaxial defects and those which cannot be classified into the first two groups [1]. Miller syndrome (OMIM 263750) is a recessively inherited AFD, which results from pyrimidine biosynthesis defects due to mutations in *DHODH* [2]. Weyers AFD (OMIM 193530) is dominantly inherited phenotype caused by mutations in either *EVC*1 or *EVC2*, leading to defective cilia-mediated hedgehog signaling [3,4]. Dominant mutations affecting the RNA polymerase subunit POLR1A and ribosomal biogenesis have been found in AFD, Cincinnati type (OMIM 616462) [5]. Related mandibulofacial dysostosis phenotypes, including Treacher-Collins syndrome, can also result from defects in ribosomal biogenesis [6]. *EFTUD2* encodes a GTPase which is a component of the U5 snRNP. Identification of *EFTUD2* mutations in mandibulofacial dysostosis of the Guion-Almeida type (OMIM 610536) demonstrated that pre-mRNA splicing abnormalities could produce an AFD [7]. Also demonstrating a link with pre-mRNA splicing, mutations in *EIF4A3* which affect the activity of the exon junction complex have been characterized in Richieri-Costa-Pereira syndrome (OMIM 268305) [8]. Similarly, mutations in the gene encoding the SF3B4 protein (also known as SAP49), a component of the U2 snRNP, have been found to produce Nager syndrome (OMIM 154400) [9–12]. While the genes involved in the characterized AFDs demonstrate that defects in a variety of basic biochemical processes can lead to these phenotypes, the underlying reasons for the distinct distribution of abnormalities in the craniofacies and distal limbs are not well understood.

Rodriguez syndrome (OMIM 201170) is a severe, usually perinatal lethal AFD characterized by severe retrognathia, hypertelorism with deep set eyes and deficient supra-orbital ridges, low set and posteriorly rotated ears, and oligodactyly with additional preaxial or postaxial abnormalities. Lower limb abnormalities, including fibular hypoplasia and equinovarus, are common and the spine, ribs and pelvis can also be involved. The initial description of the phenotype was a sibship of three affected offspring, suggesting recessive inheritance, but the seven cases reported subsequently were all sporadic in their families and did not recur, a pattern more consistent with *de novo* dominant mutations [13–19].

Recently, McPherson *et al*. described a case of a surviving child with a phenotype intermediate between Rodriguez syndrome and the non-lethal Nager syndrome form of AFD [14]. The child was heterozygous for a mutation in *SF3B4*, supporting dominant mutations as underlying the phenotype and raising the possibility that these two AFDs are allelic. To test this hypothesis, we determined the sequence of *SF3B4* in one case of classic Rodriguez syndrome and in a set of monozygotic twins with an intermediate phenotype, identifying heterozygosity for *SF3B4* mutations in both families. At the biochemical level, the mutations led to reduced SF3B4 synthesis. Cartilage RNA-seq analysis identified reduced expression and/or altered splicing of target genes, including transcription factors known to be important in skeletal development, consistent with differential effects on pre-mRNA splicing and gene expression as underlying mechanisms of disease.

## Results

### Clinical findings

#### Family R14-123

**Fig 1:**
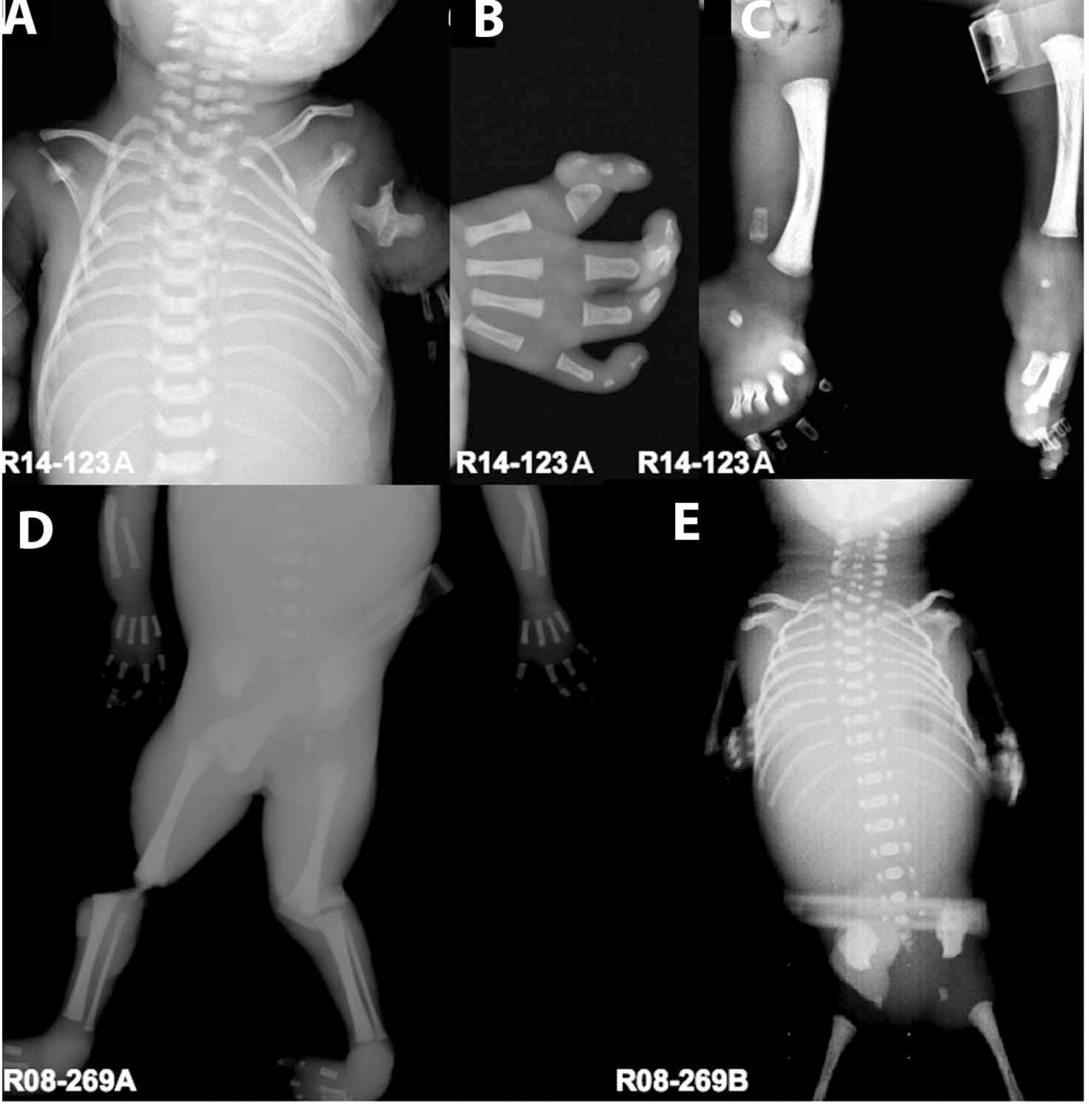
Radiographic phenotypes of cases R14-123A and R08-269A & B. (A) A/P radiograph of the chest of R14-123^a^ showing small scapulae, 11 ribs, and abnormally formed hypoplastic humeri with radioulnar synostosis. (B) Hand radiograph showing oligodactyly, hypoplastic carpal bones and preaxial polydactyly. (C) Bilateral lower extremities showing hypoplastic or absent fibulae with small stippled calcanei. (D) A/P radiograph of R08-269A showing hypoplastic radii, oligodactyly, absent thumbs, thin fibulae, and club foot. (E) A/P radiograph of R08-269B showing 11 ribs, absent radii and ulnae.

The proband, International Skeletal Dysplasia Registry (ISDR) reference number R14-123A, was born to an unaffected 26-year-old G_1_P_0_ mother and 32-year-old unaffected father. Prenatal ultrasound at 29 weeks gestation indicated that the fetus was small for gestational age and had microcephaly, severe retrognathia, severely shortened and malformed appendicular bones, oligodactyly with prexial polydactyly, coarctation of the aorta and polyhydramnios (S1 Fig and Table 1), consistent with Rodriguez syndrome, a severe acrofacial dysostosis. Preterm labor and delivery ensued at 30 weeks gestation and the newborn survived for one hour. Postnatal findings showed multiple craniofacial and distal limb abnormalities including hypertelorism, bilateral microtia, severe micrognathia, severe rhizomelia, mesomelia and oligodactyly with duplication of the thumb. Radiographs demonstrated handlebar clavicles, small scapulae, hypoplastic humeri, humeroradial synostosis, absent pubis bone, malformed radii, absent ulnae, absent fibulae and oligodactyly with preaxial polydactyly on one hand, and oligodactyly on the other hand (Fig 1 and Table 1). The clinical findings are summarized in Table 1, and, along with the perinatal lethality, supported a diagnosis of Rodriguez syndrome.

**Table 1:** Summary of the ultrasound, clinical and radiographic findings.

Case Number	R08-269A	R08-269B	R14-123
**Gestational age at delivery**	Pregnancy interrupted at 22 weeks	Pregnancy interrupted at 22 weeks	Preterm labor and delivery at 30 weeks. Died one hour after delivery
**Prenatal Ultrasound**	Craniofacial: microcephaly, micrognathia,	Craniofacial: microcephaly, micrognathia	Craniofacial: low-set deficient ears and severe retrognathia
	Extremities: shortened humeri, radii, ulnae	Extremities: shortened humeri, radii, ulnae	Extremities: severe rhizomelia of humeri, absent radii and ulnae, oligodactyly with preaxial duplication of the thumbs, femoral rhizomelia, absent fibulae, clubfeet with right oligodactyly
	Other: placenta–monochorionic/diamniotic size discordance (twin A > twin B)	Other: placenta -monochorionic/diamniotic, size discordance (twin A > twin B)	Other: polyhydramnios, coarctation of the aorta
**Postmortem Examination**	Craniofacial: hypertelorism, low-set ears and severe microtia, prominent nasal bridge, long phitrum, thin upper lip, malar hypoplasia with severe micrognathia	Craniofacial: hypertelorism, microtia, prominent nasal bridge, malar hypoplasia with severe micrognathia	Craniofacial: hypertelorism, low-set ears, microtia, broad nasal bridge, macrostomia
	Extremities: left equinovarus	Extremities: right equinovarus	Extremities: severe rhizomelia, oligodactyly of the hands with preaxial duplication of the thumbs, oligodactyly of the feet
			Other: sacral dimple
**Radiographic Findings**	Craniofacial: mandibular hypoplasia	Craniofacial: mandibular hypoplasia	Craniofacial: midface hypoplasia with marked micrognathia
	Axial:11 ribs	Axial: 11 ribs handlebar clavicles, hypoplastic scapulae, multiple cervical hemivertabrae, small ischia	Axial:11 ribs handlebar clavicles, hypoplastic scapulae, delayed ossification of the pubis
	Extremities: hypoplastic radii, thumb hypoplasia, oligodactyly, shortened diaphyses of radii	Extremities: absent/ hypoplastic radii, thumb hypoplasia, oligodactyly, shortened diaphyses of radii, cervical vertebral anomalies	Extremities: brachydactyly, triphalangeal thumb with hypoplastic middle phalange, hypoplasia of humeri, humeroradial synostosis, long radii with thickened diaphyses, ulnar aplasia, bilateral hip dislocation, fibular aplasia, delayed ossification of the talus
			Other: 11 ribs, sternal hypoplasia, abnormal ossification of the pelvis

#### Family R08-269

The twin probands (ISDR cases R08-269A and B) were the offspring of a 33-year-old unaffected mother and an unaffected father. A prenatal ultrasound at 21 weeks gestation showed a monochorionic, diamniotic pregnancy, with both twins demonstrating limb abnormalities. There was significant size discordance between the twins, but both exhibited microcephaly, micrognathia and significant shortening of the appendicular bones, consistent with a form of acrofacial dysostosis. The pregnancy was interrupted at 22 weeks gestation. Postmortem findings confirmed the size discordance, facial dysmorphisms (Fig 1), bilateral microtia, severe micrognathia, left equinovarus in twin A, and right equinvarus in twin B (Table 1) also observed on ultrasound. Radiographic abnormalities included small scapulae, hypoplasia of the radii and thumbs in both twins, oligodacytly in twin A, and cervical vertebral and rib anomalies in twin B (Fig 1 and Table 1). The clinical findings were overall less severe than classic Rodriguez syndrome, including case R14-123, but more severe than would be expected for Nager syndrome, leading to a diagnosis intermediate between Nager and Rodriguez syndromes.

### SF3B4 mutations cause Rodriguez syndrome

**Fig 2:**
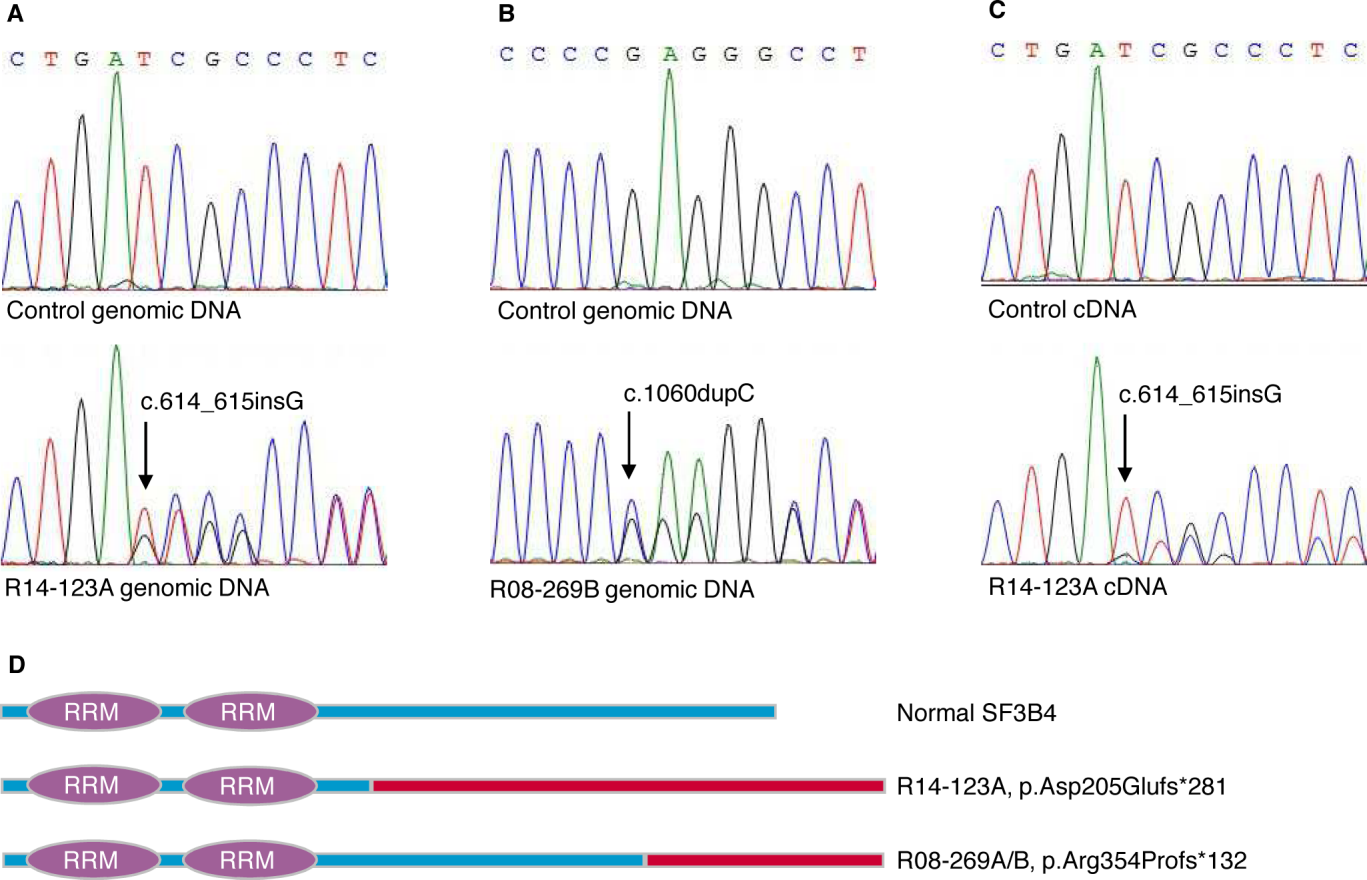
Mutations in *SF3B4*. (A,B) Electropherogram representation of genomic DNA fragments from controls (top), (A) case R14-123A (bottom), (B) case R08-269B (bottom). (C) The insertion in the *SF3B4* cDNA of R14-123A (bottom) as compared with control (top). The positions of the insertion mutations are indicated by arrows. (D) Schematic diagram of predicted protein alterations caused by *SF3B4* frameshift mutations. The blue bars correspond to the reference amino acid sequence and the red bars indicate altered amino acid sequences that begin at the mutation site. RNA recognition motifs (RRM) are shown as purple ovals.

As *SF3B4* is frequently mutated in patients with Nager syndrome and it had been suggested that Rodriguez syndrome might be an allelic disorder [9–12,14,20], we determined the sequences of *SF3B4* in the two cases. Case R14-123A, with Rodriguez syndrome, was heterozygous for a *de novo* single base insertion (c.614_615insG; p.Asp205Glufs*281) in exon 3 (Fig 2A). Case R08-269B, with the intermediate phenotype, was heterozygous for a single base insertion (c.1060dupC; p.Arg354Profs*132) in exon 5 (Fig 2B). The latter mutation was previously found in two cases of Nager syndrome [9,12]. Both mutations led to frameshifts which started at different positions within the coding region but resulted in stop codons that terminated at the same position, which was beyond the normal stop codon (Fig 2D). Such mutations are referred to as a type of nonstop mutation and can lead to nonsense mediated decay of the transcripts [21,22]. Sequence analysis of amplified cDNA from R14-123A fibroblasts showed presence of some transcript derived from the mutant allele but always at a reduced level relative to the normal allele sequence (Fig 2C), consistent with instability of the mutant transcript. Based on these findings, we concluded that *SF3B4* mutations cause Rodriguez syndrome.

### Decreased expression of SF3B4 transcript and protein in Rodriguez syndrome

**Fig 3:**
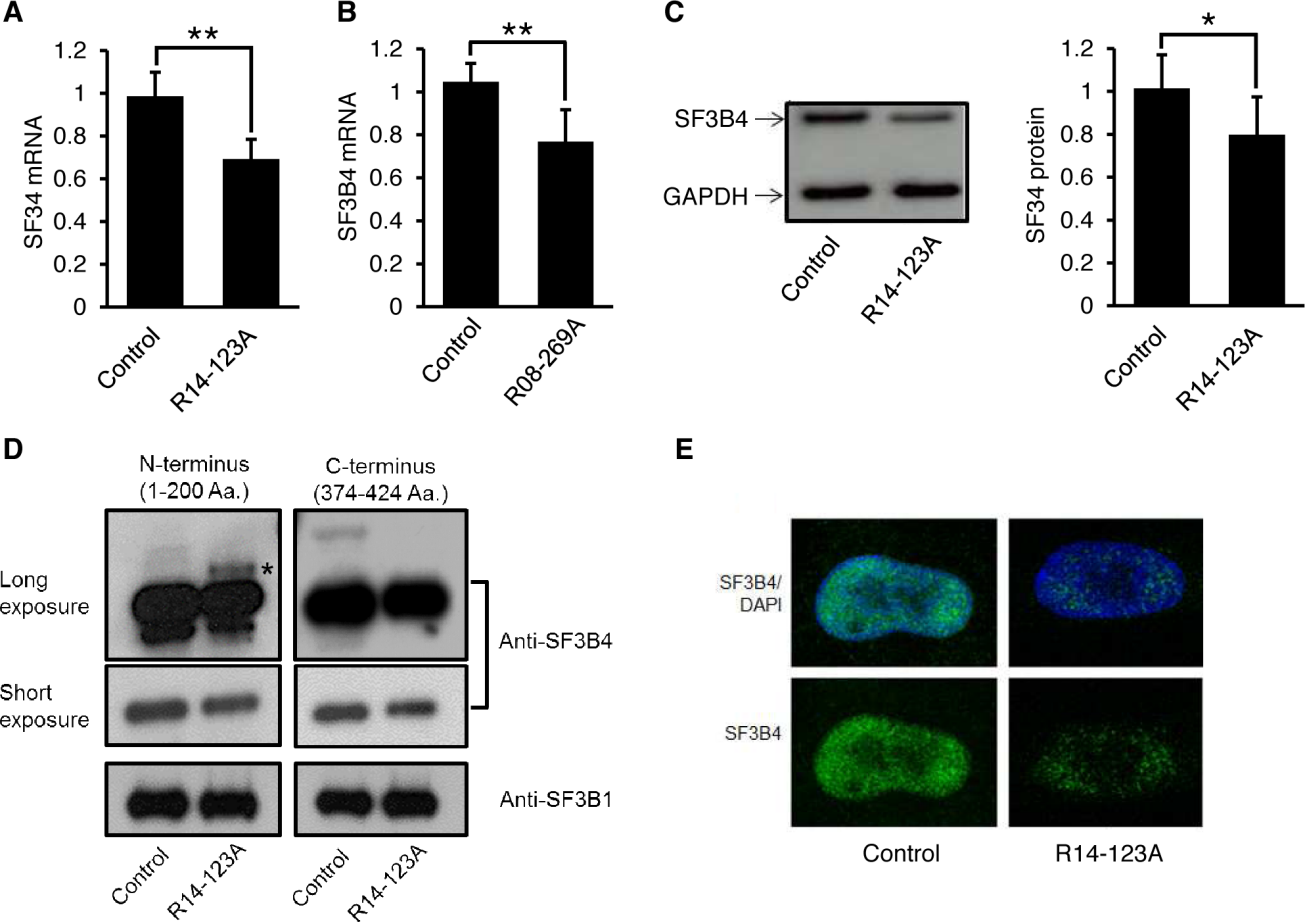
Decreased expression of SF3B4 in Rodriguez syndrome. (A,B) Detection of *SF3B4* mRNA by quantitative RT-PCR in (A) R14-123A fibroblasts and (B) R08-269A chondrocytes. (C) Western blots (left panel) and signal quantification (right panel) of SF3B4 in R14-123A fibroblasts. Data in the bar graphs were collected from at least three replicates and were represented as mean ± standard deviation. *p<0.05; **p<0.01. (D) Detection of SF3B4 protein in R14-123A fibroblasts by Western blotting with the antibodies against the N- and C-termini of the SF3B4 protein, respectively. The asterisk identifies the elongated form of SF3B4 that was presumed to be derived from the mutant allele. SF3B1 protein was used as a loading control. (E) Immunostaining of human fibroblasts with anti-SF3B4 (green). Cell nuclei were stained with DAPI (blue).

To characterize the effect of the mutations on overall *SF3B4* expression, qRT-PCR was used to measure *SF3B4* mRNA levels in cultured fibroblasts from case R14-123A and in chondrocytes from case R08-269A. There was about a 30% reduction in SF3B4 transcripts in both cases compared with controls (Fig 3A and 3B). As shown in Fig 3C, for R14-123A there was a corresponding decrease in SF3B4 protein relative to the control. Because there was a low level of the mutant transcript, which is predicted to encode a protein with an altered carboxyl-terminus that extends the length of the protein by 60 amino acids, we attempted to detect the mutant protein. The predicted molecular weight of the elongated protein is 52.8 kDa, which is 8.4 kDa larger than wild-type (WT) SF3B4. Western blotting was performed using an antibody to the amino-terminal end of the protein, which should be unaltered in both normal and mutant SF3B4, and with an antibody directed against the carboxyl-terminal end, which should only recognize WT SF3B4. As shown in Fig 3D, with a long exposure, the antibody against the amino-terminal end of the protein detected a faint protein migrating just above the SF3B4 WT protein, in a position corresponding to the predicted molecular weight of the mutant protein. This protein was not detected by the carboxyl-terminal antibody, as would be expected based on the mutation. The data were thus consistent with synthesis of a small amount of the mutant protein by the low level of mutant transcript present in the cultured cells (Fig 2C). Whether the abnormal protein is functional and contributes to the U2 snRNP and the Rodriguez syndrome phenotype is unknown. Immunofluorescence staining of cultured fibroblasts revealed nuclear localization of SF3B4, in agreement with its spliceosomal function in nucleus, with the fluorescence intensity appropriately reduced in cultured cells from the affected individual (Fig 3E).

### Disruption of growth plate architecture due to SF3B4 mutation

**Fig 4:**
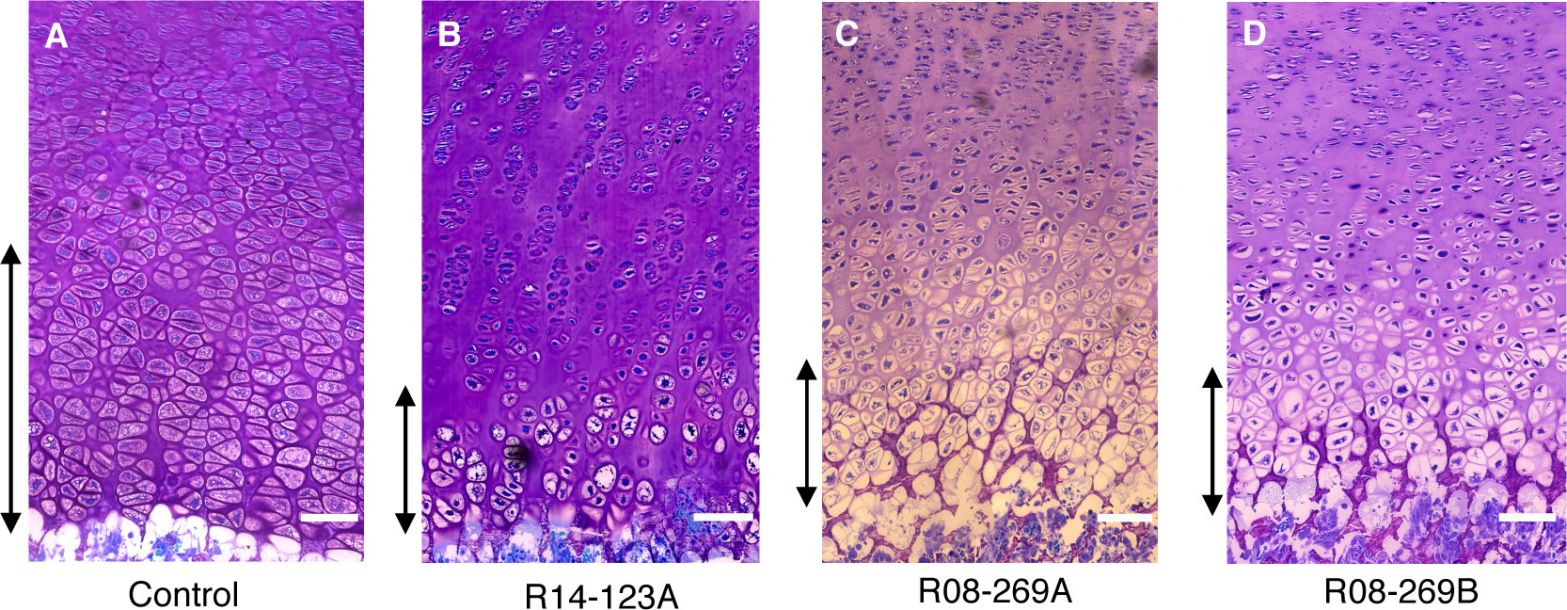
Pronounced disorganization of hypertrophic chondrocytes caused by *SF3B4* mutations. Toluidine blue staining of the distal femur growth plate from (A) a control fetus, (B) R14-123A, (C) R08-269A and (D) R08-269B. The hypertrophic zone is marked with double arrows. Images were obtained at 20X magnification. Scale bars are 100 μM.

The affected individuals exhibited shortening or absence of humeri, radii and ulnae, brachydactyly, and delayed ossification of the talus (Fig 1 and Table 1), which all indicate defective endochondral ossification. We therefore performed histopathological examination of the distal femur growth plates. As shown in Fig 4, both cases showed a dramatically reduced hypertrophic zone with disorganization of the normal columnar arrangement of chondrocytes. Mutant hypertrophic chondrocytes were also smaller than the corresponding control cells. Case R14-123A had the most severely disrupted morphology, in agreement with the more severe clinical and radiographic phenotype in this case.

### SF3B4 mutation dysregulates expression of skeletal developmental genes

**Fig 5:**
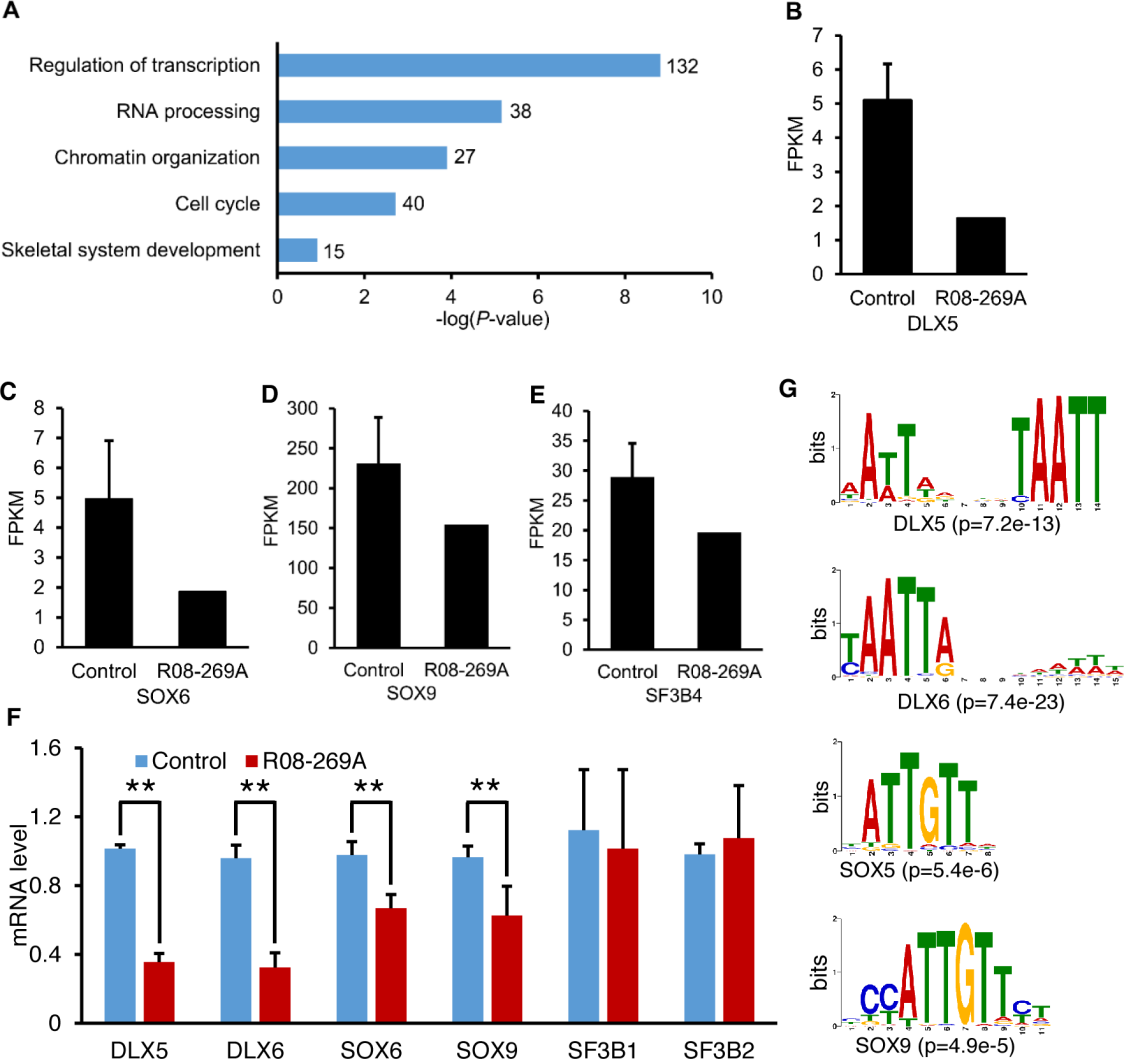
SF3B4 mutation leads to altered gene expression. (A) GO analysis of down-regulated genes in growth plate chondrocytes from case R08-269A. The number of genes in each GO term is indicated. (B-E) Expression changes of (B) *DLX5*, (C) *SOX6*, (D) *SOX9* and (E) *SF3B4*. The gene expression is represented as FPKM values. (F) Real-time quantitative RT-PCR validation of the RNA-seq data. mRNA expression was normalized to *GAPDH*. **p<0.01. (G) From top to bottom, enriched *DLX5*, *DLX6*, *SOX5* and *SOX9* binding motifs among the promoter regions of the down-regulated genes. The p values for motif enrichment are shown in parentheses below each motif.

Cartilage growth plate development is regulated by highly orchestrated cell signaling and transcriptional networks. Profound disorganization of the hypertrophic zone suggested impaired chondrocyte gene expression as a possible result of mutations in *SF3B4*. We therefore measured growth plate chondrocyte gene expression by RNA-seq using RNA derived from distal femur cartilage from case R08-269A. This analysis identified 2025 differentially expressed transcripts. Among them, 652 transcripts (representing 622 genes) were down-regulated and 1373 transcripts (representing 1215 genes) were up-regulated (S2 Table). Gene ontology (GO) analysis of the down-regulated transcripts primarily revealed altered expression related to skeletal development, transcriptional regulation, chromosome organization and cell cycle control (Fig 5A). Among the down-regulated genes, there were 15 genes, including *DLX5* and *SOX6*, that are known key regulators of skeletal development (Table 2, Fig 5B and 5C). Since *SOX9* is is an upstream regulator of *SOX6* and plays a pivitol role in chondrocyte differentiation, we examined its expression in the RNA-seq data. As shown in Fig 5D, *SOX9* is highly expressed in growth plate chondrocytes and its expression was reduced by 33% in the mutant growth plate. Thus while *SOX9* did not meet the screening criteria used to identify differentially expressed genes, its reduced expression was consistent with the secondary reduction of *SOX6* transcript levels observed in the RNA-seq data. The *SF3B4* transcript level was reduced by 32% in the mutant growth plate (Fig 5E), a result concordant with the quantitative RT-PCR data (Fig 3B).

**Table 2:** Skeletal developmental genes with reduced expression in R08-269A.

Gene symbol	RefSeq transcript ID	Log2 fold-change
*FGFR1*	NM_015850, NM_023105	-3.416721096
*IHH*	NM_002181	-2.413989037
*PRDX1*	NM_181696	-2.160981972
*COL11A2*	NM_001163771	-2.154836698
*SMAD1*	NM_005900	-1.916626325
*NOG*	NM_005450	-1.900681894
*DLX5*	NM_005221	-1.711034402
*HOXA6*	NM_024014	-1.696180867
*PKD1*	NM_000296	-1.609477294
*SOX6*	NM_033326, NM_001145811	-1.395398328
*CHAD*	NM_001267	-1.301461477
*EVC*	NM_153717	-1.197830659
*PLEKHA1*	NM_001001974	-1.146495803
*GLI2*	NM_005270	-1.016538107
*ATP7A*	NM_000052	-1.011805878

To validate the RNA-seq data, we performed quantitative RT-PCR using the same mutant chondrocyte-derived RNA. The data confirmed the observed reduction in *DLX5*, *SOX6*, and *SOX9* transcript levels (Fig 5F). The expression levels of other U2 snRNP components, *SF3B1* and *SF3B2*, did not show significant differences relative to control chondrocyte RNA. Because it has been shown that *DLX6* coordinates with *DLX5* in the regulation of craniofacial and limb development, we also examined *DLX6* expression using quantitative PCR. While *DLX6* had a low expression level in chondrocytes, and therefore did not meet inclusion criteria for determining whether it was differentially expressed in the RNA-seq analysis, by qRT-PCR its expression was significantly decreased in mutant growth plate derived cartilage RNA (Fig 5F).

DLX5/6 proteins and SOX5/6/9 are transcription factors known to regulate chondrocyte gene expression and skeletogenesis [23,24]. SOX5 and SOX6 form dimers and recognize sequences with SOX and SOX-like binding sites [25,26]. We therefore asked whether their targets were enriched among the 652 down-regulated transcripts in SF3B4 mutant growth plate chondrocytes. As shown in Fig 5G, DLX5, DLX6, SOX5 and SOX9 binding motifs were found to be significantly enriched among the promoter regions of the downregulated transcripts (S2 Table), consistent with reduced target gene expression as a regulatory consequence of reduced expression of DLX5/6 and SOX6/9 proteins.

**Fig 6:**
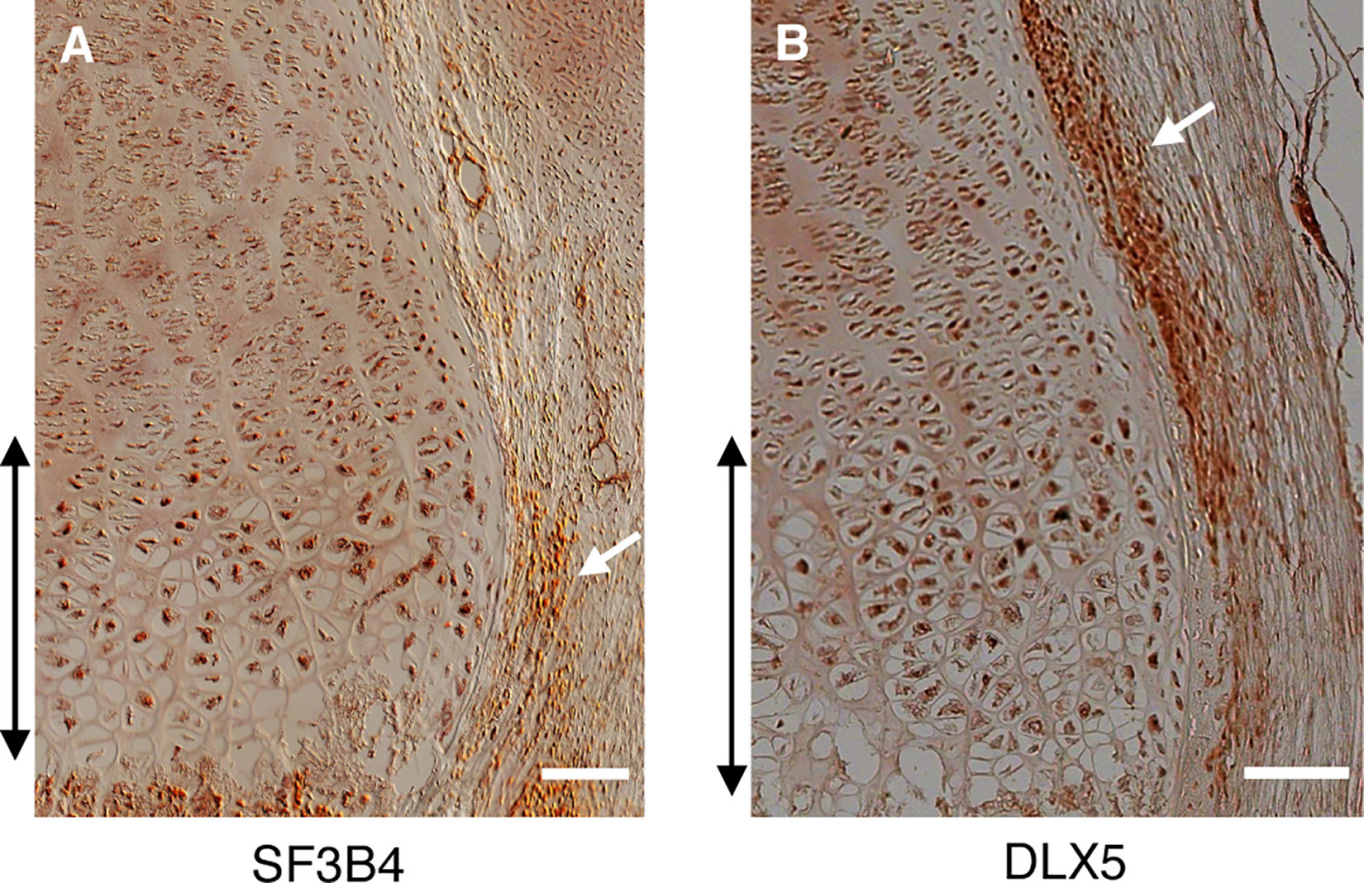
SF3B4 and DLX5 are co-expressed in the human growth plate. Immunohistochemical staining of the distal femur growth plate from a control fetus with (A) anti-SF3B4 and (B) anti-DLX5 antibodies. The hypertrophic zone is marked with double arrows. The periosteum is identified by white arrows. Images were obtained at 20X (SF3B4) and 10X (DLX5) magnification. Scale bars are 100 μM.

Immunohistochemical staining demonstrated co-localization of expression of SF3B4 and DLX5 in normal human fetal growth plate, with expression in all growth plate chondrocytes and overlapping regions of the periosteum, consistent with their roles in growth plate chondrocyte differentiation and cell signaling (Fig 6). Concordant with their respective functions in splicing and transcription, SF3B4 and DLX5 were specifically localized in chondrocyte nuclei in both wildtype (Fig 6) and mutant growth plates.

### Altered RNA splicing in SF3B4 mutant chondrocytes

**Fig 7:**
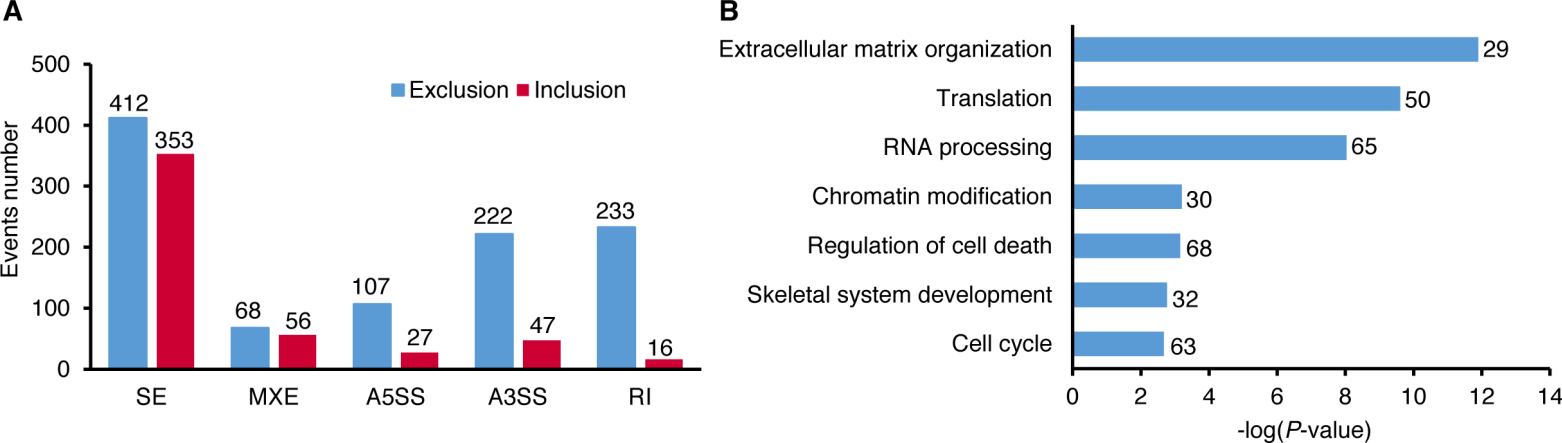
SF3B4 mutation leads to altered splicing. (A) Bar graph representation of altered splicing events in case R08-269A. The number of events in each category is indicated, with exon exclusion to the left (blue bars) and exon inclusion to the right (red bars). (B) GO enrichment analysis of alternative splicing events. The number of genes in each GO term is shown. SE, skipped exon; MXE, mutually exclusive exon; A5SS, alternative 5’ splice site; A3SS, alternative 3’ splice site; RI, retained intron.

Because of the role SF3B4 plays in the U2 snRNP complex, the rMATS algorithm was applied to the RNAseq data to determine the effect of reduced SF3B4 on splicing [27]. A total of 1541 significant differential alternative splicing events were identified, which included both exon exclusion (ΔPSI<0) and exon inclusion (ΔPSI>0) scenarios in case R08-269A compared with the controls. These events were divided among five different alternative splicing outcomes. The major effect was exon skipping, with 765 skipped exon (SE) events, including 412 exon exclusions and 353 exon inclusions (Fig 7A and S3 Table). The other four outcomes were 124 mutually exclusive exon (MXE), 134 alternative 5’ splice site (A5SS), 269 alternative 3’ splice site (A3SS) and 249 retained intron (RI) events, with a higher fraction of exon exclusions relative to inclusions (Fig 7A).

RPBJ, HIF1A, SMAD3, and IGF2R are known upstream regulators of *SOX9* expression. For all four genes, a minor proportion of their transcripts showed altered splicing, primarily exon skipping or activation of cryptic splice sites (S2 Fig). Among these events, we focused on the abnormal splicing pattern for *SMAD3*, as its altered splicing was consistent with the observed reduced expression of *SOX9*. SMAD3 is a component of TGF-β signaling pathway and induces primary chondrogenesis when overexpressed in human mesenchymal stem cells (MSCs). SMAD3 also has been shown to associate with SOX9 on the *COL2A1* enhancer to promote *SOX9*-mediated transcriptional activity, and depletion of *SMAD3* in MSCs by siRNA reduces *SOX9* expression [28,29]. In case R08-269A, a proportion of *SMAD3* transcripts had either inclusion of exon 3 or increased transcription that started from exon 4. Exon 3 encodes a linker region that connects the MH1 and MH2 domains of SMAD3. The *SMAD3* splicing variant lacking exon 3 (*SMAD3-Δ3*) is expressed in multiple human tissues and has positive transcriptional activity [30]. The truncated *Smad3* isoform transcribed from an ATG site within exon 4 consists of 7 exons and encodes half of the linker region and the MH2 region. This isoform inhibits activin-induced activation of the FSHβ promoter [31]. Thus together the R08-269A transcripts had reduced expression of the *SMAD3-Δ3* transcription activator and increased expression of 5’ truncated transcriptional repressor isoforms, consistent with reduced SMAD3 activity which could lead to reduced expression of *SOX9*.

Beyond these specific splicing changes, GO analysis of the overall differential alternative splicing events demonstrated enrichment of biological processes relevant to the SF3B4 mutant phenotype, including extracellular matrix organization, skeletal system development, and RNA processing (Fig 7B). These data support a complex effect of altered RNA splicing on downstream gene expression that included signaling molecules, transcription factors and chromatin modifiers that are known to regulate genes essential for skeletal development. Our findings imply that these factors could contribute to the mechanism of disease, but how each molecule individually contributes to this complex phenotype will require development of tissue specific cellular and animal models.

## Discussion

Spliceosomes, large ribonucleoprotein complexes that remove introns from pre-mRNA molecules, are composed of a set of snRNPs, including the U1, U2, U4/U6, and U5 complexes. SF3B4 encodes a core protein in the mammalian SF3B complex, part of the U2-type spliceosome which acts to help tether the U2 snRNP to the branch site [32]. The *SF3B4* mutations identified lead to reduced SF3B4 synthesis, altered pre-mRNA splicing and reduced gene expression, ultimately producing the defects in craniofacial and limb development observed in the SF3B4 disorders.

### *SF3B4* haploinsufficiency and AFD

Haploinsufficiency for *SF3B4* is considered to be the major cause of Nager syndrome. In an analysis of 35 independent families with Nager syndrome, Bernier et al. identified 18 cases heterozygous for *SF3B4* mutations, including 14 frameshift and two nonsense mutations, indicating that dominant, loss-of-function mutations commonly produce the phenotype [9]. Similarly, in the data presented here, two *SF3B4* mutations that were both predicted to result in SF3B4 molecules with altered and extended carboxyl-termini, produced similar but more severe acrofacial dysostosis phenotypes, including one case with the lethal Rodriguez syndrome phenotype. Such mutations are predicted to lead to nonsense-mediated decay of the transcript and in the one case examined the mutant transcript was largely degraded. Whether the small amount of remaining mutant mRNA and the small amount of the abnormal protein (Fig 3D) synthesized by the cells could exert a dominant-negative or gain-of-function effect on the SF3B complex and contribute to the phenotype by altering the splicing function of the complex is unknown. However, it is noteworthy that a case of Nager syndrome with a *de novo* deletion at chromosome 1q21.2 that covers *SF3B4* has been described [33], supporting *SF3B4* haploinsufficiency as the primary driver of the craniofacial and limb defects observed in these acrofacial dysostosis phenotypes. The clinical variability in expression across Nager syndrome cases with defects in SF3B4 does suggest that modifiers can influence the phenotype, so it may be that small differences in splicing activity could contribute measureably to phenotypic expression. Interestingly, even the monozygotic twins described here showed some variability in expression, with twin A exhibiting a more severe clinical and radiographic phenotype, suggesting that either epigenetic phenomena or small temporal developmental differences in gene expression can alter the clinical consequences of *SF3B4* mutations.

### Transcriptional dysregulation by SF3B4 mutation

The RNA-seq data demonstrate that reduced SF3B4 activity can alter gene expression, as in growth plate chondrocytes there were 622 genes that showed reduced expression. Among these are genes known to regulate skeletal system development, consistent with the altered growth plate morphology observed in *SF3B4* phenotypes and the ongoing growth deficiency in these disorders. Particularly noteworthy were *DLX5* and *SOX9*, genes with reduced expression and for which mechanistic hypotheses can be advanced to explain some of the skeletal phenotypic features observed in the SF3B4 disorders.

*DLX5* and its cis-linked paralogue *DLX6* are pivotal regulators of craniofacial and limb development. *Dlx5* knockout mice showed craniofacial abnormalities [34] and *Dlx5/6*^−/−^ mutant embryos exhibited severe limb, craniofacial, and axial skeletal defects [35,36]. *Dlx5*^−/−^ mice showed a modest defect in chondrocyte hypertrophy in the long bones of the limbs [37], while targeted deletion of both *Dlx5* and *Dlx6* resulted in more pronounced deficiencies in chondrocyte hypertrophy [36], underscoring their co-regulatory roles in growth plate chondrocyte maturation and endochondral ossification. Reciprocally, forced overexpression of *Dlx5* in chondrocytes promoted hypertrophy and induced precocious ossification in the endochondral skeleton [38,39].

In humans, chromosomal rearrangements of the 7q21.3-q22.1 region harboring *DLX5*, *DLX6* and their regulatory elements (including enhancers) have been found in Split-hand/split-foot malformation 1 (SHFM1, OMIM 183600), indicating that structural abnormalities affecting both genes can produce SHFM1 [40,41]. Monoallelic deletion of only the *DLX5/6* locus in some cases indicated that *DLX5/6* haploinsufficiency can lead to SHFM1 [42]. Point mutations in three SHFM1 families suggest that mutations in *DLX5* alone may be sufficient to produce the phenotype [43–45]. These data support the idea that the approximately half normal *DLX5/6* expression resulting from *SF3B4* haploinsufficiency could produce the distal limb abnormalities observed in Nager and Rodriguez syndromes. In addition to the split-hand/split foot phenotype, SHFM1 cases can have craniofacial abnormalities, mesomelic limb malformations, hearing loss, and developmental delay. Since many of these features are shared by Rodriguez/Nager syndrome cases, including cleft palate, micrognathia, and hypoplasia/absence of the radii and fibulae, the reduction of *DLX5*/*6* expression that resulted from reduced *SF3B4* expression likely contributes to these aspects of the phenotype as well. Because *DLX5/6* transcripts were spliced normally, reduced expression of *DLX5/6* due to *SF3B4* mutation is likely to be indirect. Multiple mechanisms are known to regulate *DLX5/6* expression, including regulation by other transcription factors (e.g. *p63*, *MEF2C*) [35,46], by distal regulatory elements (enhancer and noncoding RNA) [47–51] and by epigenetic mechanisms [52,53]. *SF3B4* mutations may interfere with one or more of these mechanisms and lead to dysregulation of *DLX5/6* gene expression.

SOX9 is a transcription factor that regulates skeletal development [54–57]. Heterozygosity for mutations in *SOX9* produces campomelic dysplasia [54,55], a generally lethal skeletal dysplasia [58]. Most mutations in campomelic dysplasia result in haploinsufficiency for *SOX9* and the phenotypic consequences include micrognathia and abnormal ears, as well as distinct skeletal abnormalities. The characteristic skeletal defects include a bell shaped thorax, eleven pairs of ribs, a lateral clavicular hook, hypoplastic scapulae, poor ossification of the pubis, hip dislocations, hypoplastic fibulae, poor ossification of the talus and club feet. The cases with *SF3B4* mutations studied here exhibited many of these findings, supporting the inference that the diminished expression of *SOX9* transcript due to *SF3B4* haploinsufficiency contributes to their skeletal abnormalities. Furthermore, it is known that the skeleton is exquisitively sensitive to SOX9 dosage. Mouse studies show that haploinsuffiency produces a skeletal phenotype similar to campomelic dysplasia in that *Sox9*^*+/-*^ mice show hypoplastic scapulae and thin pubic bones [59]. Similar to the consequences of diminished *DLX5/6* expression, because *SOX9* transcripts appear to be spliced normally, the mechanism for diminished *SOX9* transcript may be indirect, but the remarkable phenotypic overlap supports the hypothesis that reduced *SOX9* expression has the predictable biological consequences in the *SF3B4* disorder phenotypes.

The disrupted hypertrophic zones of the patient growth plates (Fig 4) raise the question of whether downregulation of *SOX9* and *DLX5* are the cause or consequence of the morphological abnormalities. According to previous studies, *Sox9* is expressed in reserve and proliferating chondrocytes but not in the hypertrophic zone [26,60], consistent with an effect on hypertrophic chondrocyte differentiation as a consequence of reduced SOX9. For *Dlx5*, the gene is expressed throughout the growth plate, including in differentiating proliferative chondrocytes as well as the prehypertrophic and hypertrophic zones [37,39,61]. However, because the hypertrophic zone is much less cellular, loss of hypertrophic chondrocytes could not account for the approximately 50% reduction in *DLX5* transcripts. Thus the data again suggest that the morphological alterations are secondary to the reduced expression and not the other way around.

### Splicing defects and AFD

The altered splicing and gene expression resulting from *SF3B4* mutations echo the spliceosomal defects that have been found to underlie other craniofacial disorders [6]. Haploinsufficiency for *EFTUD2*, which encodes a GTPase in the U5 snRNP and has a regulatory role in catalytic splicing and post-splicing-complex disassembly, causes mandibulofacial dysostosis of the Guion-Almeida type (OMIM 610536), a disorder that shares many clinical features with Rodriguez/Nager syndromes [7]. EFTUD2 has been shown to interact with SF3B4 by yeast two-hybrid and co-immunoprecipitation studies and it has been suggested that interactions between SF3B and U5 proteins may recruit the tri-snRNP (U4/U6-U5) complex to the spliceosome via the U2 complex [62]. In this study, we identified 1541 alternative splicing events, predominantly exon skipping. As shown here, altered splicing has complex consequences, likely contributing both directly and indirectly to the AFD phenotype, a complexity underscored by alterations in expression of multiple transcription factors known to be essential for normal skeletal development. For the craniofacial dysmorphology in particular, knockdown of *Sf3b4* in Xenopus has been shown to result in loss of cranial neural crest cells, demonstrating the importance of normal splicing in maintaining craniofacial skeletal precursors during development [63].

The limb deficiencies and craniofacial dysmophologies found in AFDs with spliceosomal defects indicate that altered splicing/expression of key regulators may lead to developmental abnormalities including early digit patterning and neural crest cell formation and proliferation defects [64], neither of which would be revealed by studying growth plate chondrocytes. For example, *p63* is a master trancriptional regulator that controls *DLX5/6* expression and skeletogenesis [35,65,66]. *p63*^*−/−*^ mice show severely limb and craniofacial defects, and *P63* mutations lead to human disorders affecting the facial and limb structures [65–69]. Multiple *p63* isoforms generated by alternative splicing and promoter usage have been identified, which show distinct roles during endochondral ossification [70]. Therefore, mis-spliced *p63* may account for the down-regulation of *DLX5/6* in Rodriguez/Nager syndrome cases. However, *p63* expression was undetectable in growth plate chondroctyes, suggesting the possibility that earlier developmental events may have led to some of the observed effects on gene expression. Analysis of *Sf3b4* deficient animal models will help to identify novel regulators and clarify the role of *Sf3b4* in skeletogenesis at early developmental stages.

In addition to the links between splicing and AFD, other mechanisms may also contribute to the phenotype. In this context, SF3B4 has been shown to bind the bone morphogenetic protein (BMP) receptor BMPR-IA and specifically inhibit BMP-mediated osteochondral cell differentiation [71,72]. Thus reduced *SF3B4* expression could alter more than one developmental pathway to elicit the observed phenotypes. However, *Xenopus Sf3b4* knockdown studies did not confirm a link between SF3B4 expression and BMP signaling alterations [63], indicative of unresolved certainty connecting the SF3B4 disorders and this important skeletal signaling pathway.

In summary, the current study identified heterozygosity for *SF3B4* mutations in one case of Rodriguez syndrome and second case with a phenotype intermediate between Rodriguez and Nager syndromes. These data demonstrate that Rodriguez syndrome is a dominant disorder that is allelic with Nager syndrome, and show that the two disorders belong to a spectrum unified by mutations in *SF3B4*. *SF3B4* mutations have also been found in an independent set of Rodriguez syndrome cases (New dominant mutations in SF3B4 encoding an mRNA spliceosomal protein important in embryonic limb patterning underlie Rodriguez acrofacial dysostosis. MD Irving, B Dimitrov, D Chitayat, JI Rodriguez, MW Wessels, MA Simpson. American Society of Human Genetics Meeting, 2014), further supporting this conclusion. Through RNA-seq analysis, mutant growth plate chondrocytes showed altered splicing and reduced expression of transcription factors and additional genes essential for skeletogenesis, suggesting specific regulatory mechanisms that are disrupted in the *SF3B4* disorders. Elucidating the direct and indirect effects on the regulatory gene expression pathways affected by *SF3B4* mutations is expected to reveal the developmental hierarchy that defines when and how skeletogenesis is altered to produce the SF3B4 phenotypes.

## Materials and Methods

### Ethics statement

Cases were recruited and informed consent obtained under a UCLA-approved human subjects protocol, through which clinical evaluation and imaging studies, as well as tissue procurement, were carried out.

### Cartilage specimen collection and cell culture

Chondrocytes were isolated from distal femur growth plate cartilage from case R08-269A and four independent 14–18 week normal fetal growth plates as described previously [73]. Skin fibroblast cultures were established from explanted skin biopsies from case R14-123A and an unaffected 26-week fetus. Fibroblasts were grown at 37°C in Dulbecco's modified Eagle’s medium plus 10% FBS, penicillin, and streptomycin in 5% CO_2_.

### Genomic DNA isolation and Sanger sequencing

Genomic DNA was isolated from cultured fibroblasts using the QIAamp DNA Mini Kit (Qiagen) according to the manufacturer’s protocol. PCR products were generated with primers flanking each coding exon of *SF3B4* (S1 Table) and their sequences determined by bidirectional Sanger sequencing. Electropherograms were analyzed using the CLC Main Workbench (CLC Bio). DNA sequences were compared with the *SF3B4* reference genomic sequence (NCBI accession number NG_032777.1).

### RNA isolation and quantitative PCR

RNA was isolated from fibroblasts and chondrocytes with TRIZol (Thermo Fisher Scientific) and DNA contamination removed with DNase I (Thermo Fisher Scientific). RNA was reverse transcribed using the SuperScript III First-Strand Synthesis System (Thermo Fisher Scientific). Quantitative real-time PCR was performed using SYBR Green Real-Time PCR Master Mix (Thermo Fisher Scientific). Primer sequences for the target genes are listed in the S1 Table. *GAPDH* was used as a reference gene for normalization. Gene expression was quantified with the ΔΔCT method [74]. Statistical significance was assessed using two-sample Student’s *t*-test. Sequences of PCR products were determined by Sanger sequence analysis and compared with the NCBI *SF3B4* reference sequence (NCBI accession number NM_005850.4).

### Western blotting

Cells were lysed in RIPA buffer and proteins separated by electrophoresis on SDS-PAGE gels (Bio-Rad). Proteins were transferred to membranes and probed with anti-SF3B1 (Abcam, ab172634, 1:1000 dilution), anti-SF3B4 N-terminal (Proteintech, 10482-1-AP, 1:1000 dilution), anti-SF3B4 C-terminal (Bethyl Laboratories, A303-950A, 1:1000 dilution) or anti-GAPDH (Cell Signaling Technology, 2118, 1:10000 dilution) antibodies. The ECL kit (Thermo Fisher Scientific) was used to visualize immunoreactive proteins. The level of SF3B4 was quantified with ImageJ software and normalized by comparison with the expression of GAPDH.

### Cellular immunofluorescence

Cultured fibroblasts were fixed in 4% PFA, followed by three washes with PBS and one wash with PBST (1% Triton X-100 in PBS). The samples were then blocked in 10% goat serum for 1h and incubated overnight with anti-SF3B4 polyclonal antibody (Proteintech, 10482-1-AP 1:50 dilution). Cell images were captured using confocal fluorescence microscopy (Zeiss).

### Cartilage morphology and immunohistochemistry

For Toluidine Blue staining, fetal cartilage samples were fixed in 10% buffered formalin overnight, then washed and transferred to 70% ethanol, dehydrated and embedded without decalcification in methyl methacrylate. The cartilage sections were stained with 0.1% Toluidine Blue. For immunohistochemistry, the paraffin embedded sections were deparaffinized, rehydrated in an ethanol series and permeablilized in 0.5% Triton X-100 before blocking in 10% goat serum and incubating with the primary antibody. The anti-SF3B4 (Proteintech, 10482-1-AP, 1:50 dilution) and anti-DLX5 (Sigma-Aldrich, HPA005670, 1:150 dilution) antibodies were used for staining. The sections were developed with 3,3'-diaminobenzidine (DAB) solution.

### RNA-seq and data processing

Total RNA was isolated with TRIZol (Thermo Fisher Scientific) from distal femur growth plate chondrocytes from case R08-269A and four independent 14–18 week fetuses. DNA contamination was removed by DNase I (Thermo Fisher Scientific) digestion at 37°C for 30 minutes. The RNA was further purified with the RNeasy Mini Kit (Qiagen). Total RNA samples with RIN (RNA integrity) numbers ≥ 8 (measured with the Agilent 2100 BioAnalyzer) were used for mRNA library preparation using the TruSeq RNA Preparation Kit (Illumina) according to the manufacturer's instructions. 75bp paired-end reads were generated on the HiSeq 2000 or NextSeq 500 sequencer (Illumina).

RNA-seq reads were mapped to the human genome (hg19) using TopHat (v2.0.9), allowing up to 2-bp mismatches per read [75]. Cufflinks (v2.2.1) was used to calculate RNA-seq based gene expression levels using the FPKM value (fragments per kilobase of exon per million fragments mapped) [76]. Low abundance genes with FPKM values less than 1 were filtered before further analysis. The differentially expressed genes in the case were defined as those genes with more than a 2-fold change in FPKM value compared with the average FPKM value of the normal control samples and more than a 1.5-fold change of FPKM value compared with the maximum or minimum FPKM value of the controls. rMATS (v3.2.1) was used to identify differential alternative splicing events between the case and controls [27]. The change in Percent Spliced In (ΔPSI or Δψ) and false discovery rate (FDR) were used to identify the most significant altered splicing events. The alternative splicing events were filtered using |ΔPSI| > 0.05, FDR < 0.05 and at least 10 reads covering the exon-exon junctions in both case and control samples. Genes with differential expression or alternative splicing were submitted to DAVID for Gene Ontology (GO) analysis [77].

### Motif analysis

Enrichment analysis of known transcription factor binding motifs was performed with CentriMo v4.11.1 in the MEME suite [78] using the DNA sequences from gene promoter regions (±1Kb from transcription start sites). Known motifs were obtained from the HOmo sapiens COmprehensive MOdel COllection (HOCOMOCO) v10 [79]. Match score ≥ 5 and E-value ≤ 10 were selected as the report threshold.

## Supporting Information

S1 Fig Prenatal ultrasound images for case R14-123A. (A,B). Profile showing severe retrognathia and malar hypoplasia. (C) Preaxial polydactyly of the hand (arrow). (D) Low set ears (arrow). (E) Humeral-radial fusion. (F) Bilateral lower extremities showing shortened tibiae and absent fibulae.
(TIF)

S2 Fig Altered RNA splicing in growth plate chondrocytes. Sashimi plots of alternatively spliced and flanking exons of (A) *SMAD3*, (B) *IGF2R*, (C) *RBPJ*, and (D) *HIF1A*. The number of junction reads is indicated within segments. Altered splicing events are indicated by arrows. Exons are numbered and shown at the bottom.
(TIF)

S1 Table Sequences of primers used in this study. (XLSX)

S2 Table Gene expression in growth plate chondrocytes and motif analysis. (XLSX)

S3 Table Alternative splicing events in growth plate chondrocytes from case R08-269A. (XLSX)
