# Correction: Altered mRNA Splicing, Chondrocyte Gene Expression and Abnormal Skeletal Development due to *SF3B4* Mutations in Rodriguez Acrofacial Dysostosis

**DOI:** 10.1371/journal.pgen.1006502

**Published:** 2016-12-09

**Authors:** Felipe Marques, Jessica Tenney, Ivan Duran, Jorge Martin, Lisette Nevarez, Robert Pogue, Deborah Krakow, Daniel H. Cohn, Bing Li

## Notice of Republication

The original Fig. 1 contained images incompatible with *PLOS Genetics'* confidentiality standards. The error was corrected by removing the images in the HTML and PDF versions of this article on November 18^th^, 2016 and the corrected Fig. 1 is available in the supporting information of this article.

## Supporting Information

S1 File Corrected Fig. 1. Radiographic phenotypes of cases R14-123A and R08-269A & B. (A) A/P radiograph of the chest of R14-123ª showing small scapulae, 11 ribs, and abnormally formed hypoplastic humeri with radioulnar synostosis. (B) Hand radiograph showing oligodactyly, hypoplastic carpal bones and preaxial polydactyly. (C) Bilateral lower extremities showing hypoplastic or absent fibulae with small stippled calcanei. (D) A/P radiograph of R08-269A showing hypoplastic radii, oligodactyly, absent thumbs, thin fibulae, and club foot. (E) A/P radiograph of R08-269B showing 11 ribs, absent radii and ulnae.
(TIF)
