# Supplementary figures and images for: Altered mRNA Splicing, Chondrocyte Gene Expression and Abnormal Skeletal Development due to SF3B4 Mutations in Rodriguez Acrofacial Dysostosis
Source: PLoS Genet. 2016 Sep 13;12(9):e1006307. doi: 10.1371/journal.pgen.1006307 (PMC5021280; doi:10.1371/journal.pgen.1006307)

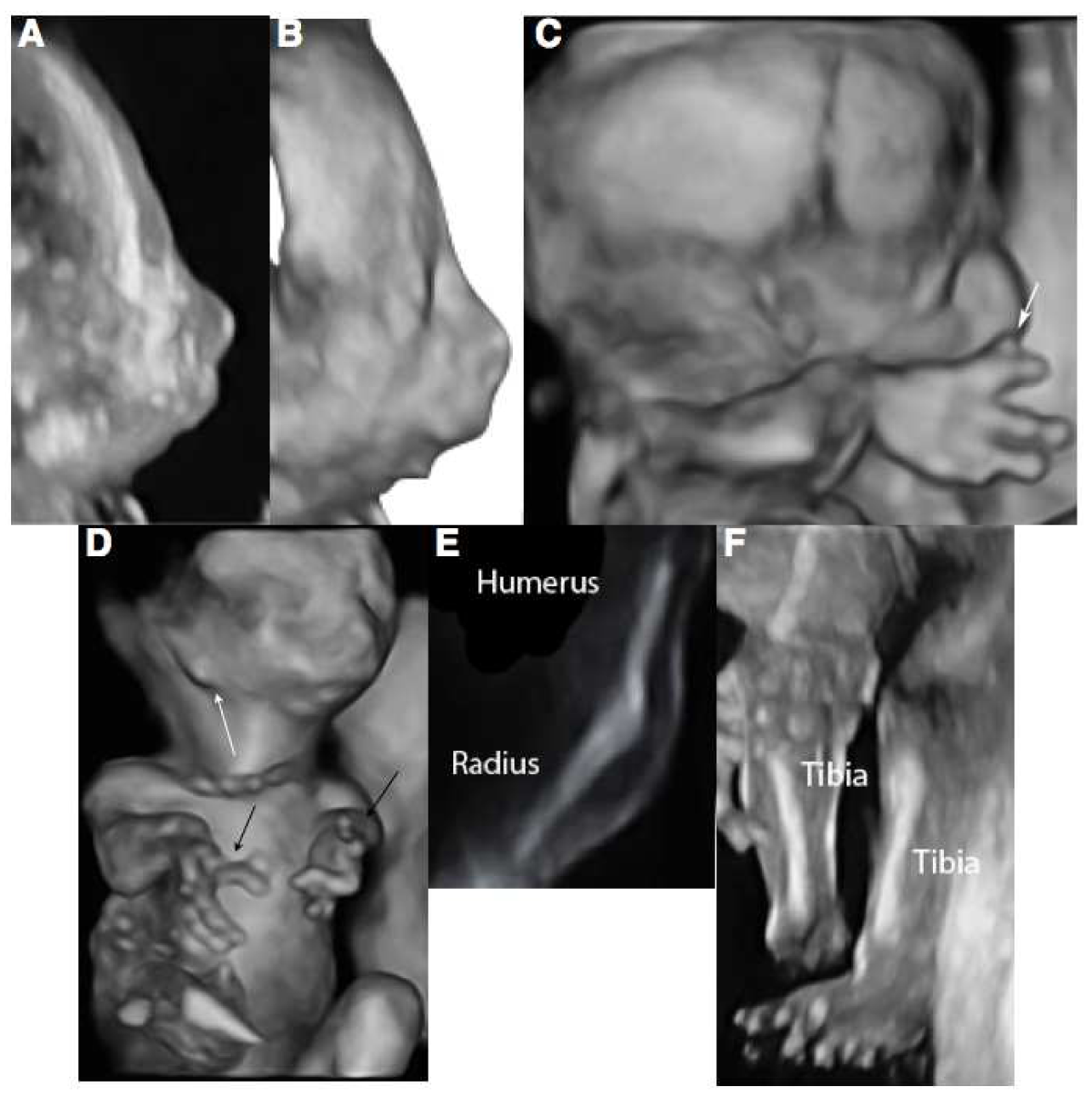

Supplement: S1 Fig — (A,B). Profile showing severe retrognathia and malar hypoplasia. (C) Preaxial polydactyly of the hand (arrow). (D) Low set ears (arrow). (E) Humeral-radial fusion. (F) Bilateral lower extremities showing shortened tibiae and absent fibulae. (TIF) [file pgen.1006307.s001.tif]

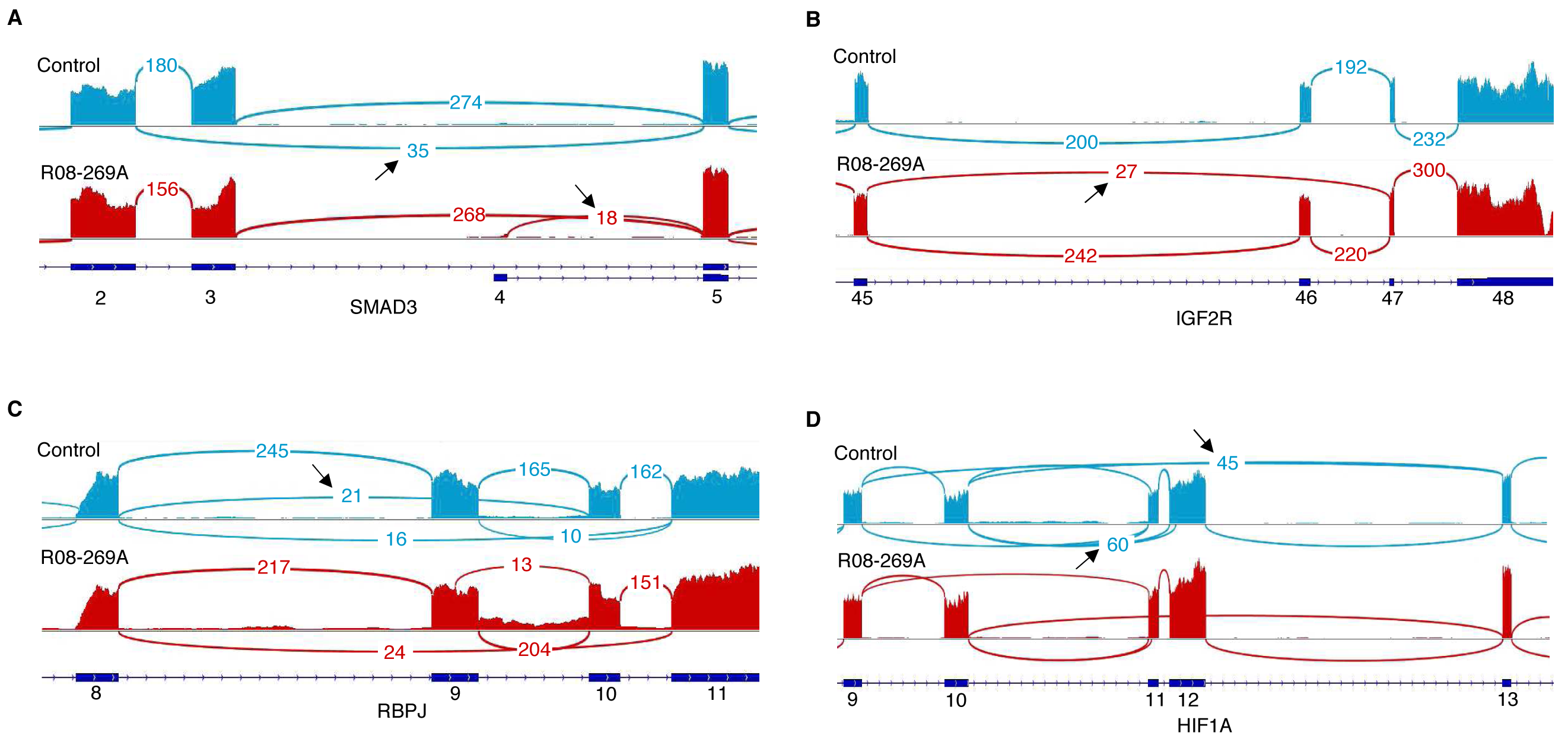

Supplement: S2 Fig — Sashimi plots of alternatively spliced and flanking exons of (A) SMAD3, (B) IGF2R, (C) RBPJ, and (D) HIF1A. The number of junction reads is indicated within segments. Altered splicing events are indicated by arrows. Exons are numbered and shown at the bottom. (TIF) [file pgen.1006307.s002.tif]
