# Supplementary figures and images for: Correction: Altered mRNA Splicing, Chondrocyte Gene Expression and Abnormal Skeletal Development due to SF3B4 Mutations in Rodriguez Acrofacial Dysostosis
Source: PLoS Genet. 2016 Dec 9;12(12):e1006502. doi: 10.1371/journal.pgen.1006502 (PMC5147806; doi:10.1371/journal.pgen.1006502)

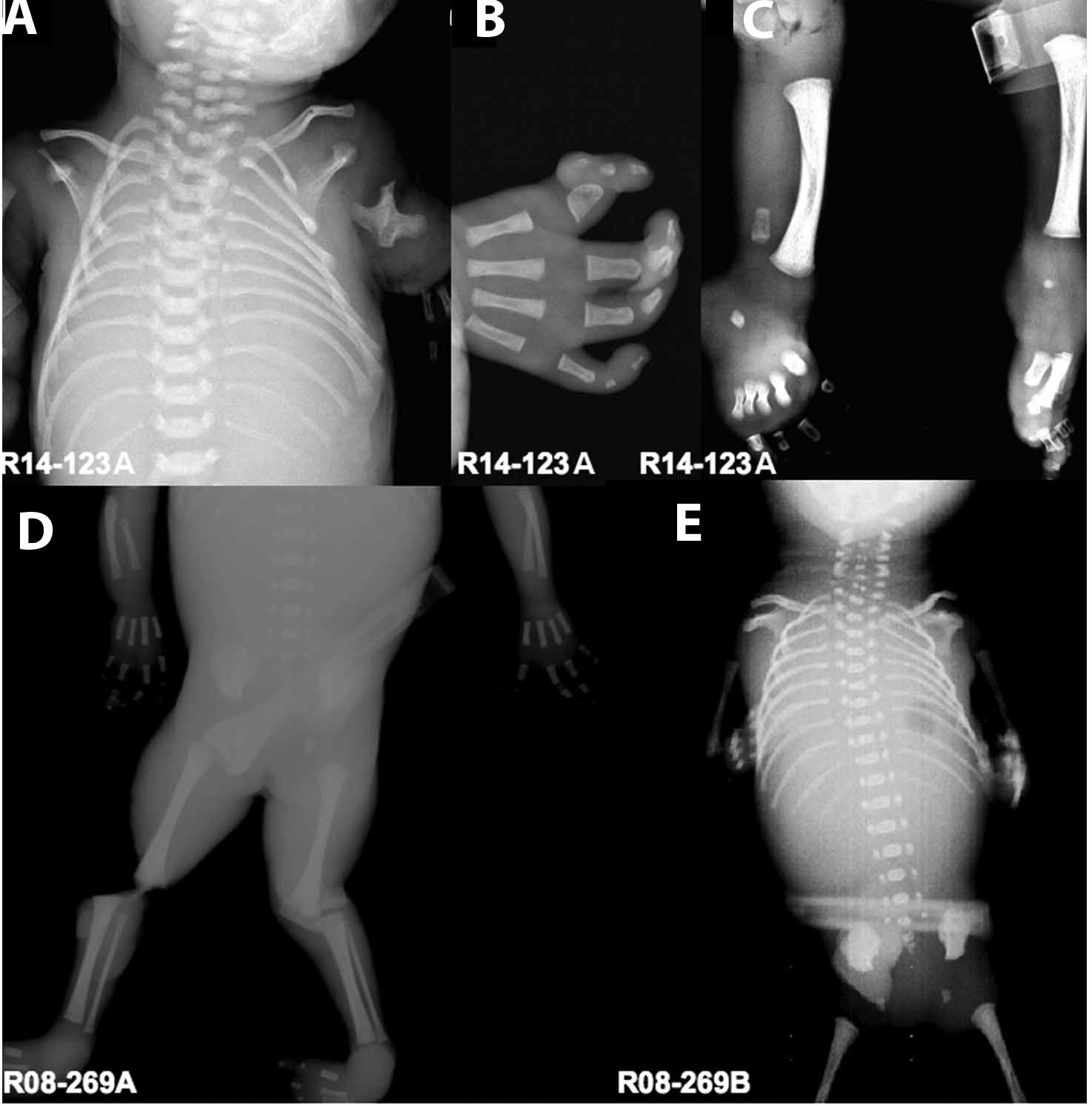

Supplement: S1 File — Radiographic phenotypes of cases R14-123A and R08-269A & B. (A) A/P radiograph of the chest of R14-123ª showing small scapulae, 11 ribs, and abnormally formed hypoplastic humeri with radioulnar synostosis. (B) Hand radiograph showing oligodactyly, hypoplastic carpal bones and preaxial polydactyly. (C) Bilateral lower extremities showing hypoplastic or absent fibulae with small stippled calcanei. (D) A/P radiograph of R08-269A showing hypoplastic radii, oligodactyly, absent thumbs, thin fibulae, and club foot. (E) A/P radiograph of R08-269B showing 11 ribs, absent radii and ulnae. (TIF) [file pgen.1006502.s001.tif]
